# Measuring thermodynamic details of DNA hybridization using fluorescence

**DOI:** 10.1002/bip.21615

**Published:** 2011-07

**Authors:** Yong You, Andrey V Tataurov, Richard Owczarzy

**Affiliations:** Department of Molecular Genetics and Biophysics, Integrated DNA TechnologiesCoralville, IA

**Keywords:** fluorophore, quencher, oligonucleotide folding, duplex stability, nucleic acid

## Abstract

Modern real-time PCR systems make it easy to monitor fluorescence while temperature is varied for hundreds of samples in parallel, permitting high-throughput studies. We employed such system to investigate melting transitions of ordered nucleic acid structures into disordered random coils. Fluorescent dye and quencher were attached to oligonucleotides in such a way that changes of fluorescence intensity with temperature indicated progression of denaturation. When fluorescence melting data were compared with traditional ultraviolet optical experiments, commonly used dye/quencher combinations, like fluorescein and tetramethylrhodamine, showed substantial discrepancies. We have therefore screened 22 commercially available fluorophores and quenchers for their ability to reliably report annealing and melting transitions. Dependence of fluorescence on temperature and pH was also investigated. The optimal performance was observed using Texas Red or ROX dyes with Iowa Black RQ or Black Hole quenchers. These labels did not alter two-state nature of duplex melting process and provided accurate melting temperatures, free energies, enthalpies, and entropies. We also suggest a new strategy for determination of DNA duplex thermodynamics where concentration of a dye-labeled strand is kept constant and its complementary strand modified with a quencher is added at increasing excess. These methodological improvements will help build predictive models of nucleic acid hybridization. © 2011 Wiley Periodicals, Inc. Biopolymers 95: 472–486, 2011.

## INTRODUCTION

Fluorescence in the visible spectrum has been used to detect hybridization of nucleic acid over two decades.^1^–^4^ Several strategies have been designed, which rely on attachment of a fluorophore (fluorescent dye) and a quencher to nucleic acid.^2^ The melting transition of duplex DNA is coupled to separation of fluorophore and quencher, so the extent of the transition is sensed from fluorescence signal. In one approach, the fluorophore and the quencher are attached at termini of a short hairpin molecule.^5^,^6^ When this structured single strand (molecular beacon) hybridizes to complementary target sequence, the hairpin structure is disrupted, fluorophore and quencher are separated, and fluorescence increases. In another approach, the one that we studied here, one strand is labeled with fluorophore and the other strand is labeled with a quencher. Annealing of strands brings the dye and the quencher in very close proximity, therefore, fluorescence of the fluorophore decreases.

Introduction of real-time PCR assays spearheaded development of equipment than can measure fluorescence for hundreds of small volume samples in parallel on plastic plates. Biophysicists have recently taken advantage of these commercially-available real-time PCR systems and employed them to determine melting temperatures (*T*_m_) of quadruplexes,^7^–^9^ molecular beacons,^5^,^6^ duplexes, triplexes,^10^ and nanostructures.^11^,^12^ However, traditional ultraviolet (UV) absorbance and calorimetric melting experiments have provided additional important information beyond melting temperatures.^13^–^15^ The nature of the melting process (two-state or non-two-state) has been evaluated, and changes in enthalpy, entropy, and free energy have been determined. These thermodynamic values are important for in silico predictions of nucleic acid hybridizations when oligonucleotide applications are designed. Thermodynamic effects are often sequence-dependent, so melting experiments are necessary for hundreds of sequences to obtain accurate parameters for a single chemical modification or structural element.^16^–^18^ Since traditional UV spectroscopic and calorimetric methods are low throughput, thermodynamic parameters and accurate *T*_m_ predictions are not available for most of useful DNA modifications, e.g., 2′-*O*-methyl RNA, locked nucleic acids, phosphorothioates. High-throughput fluorescence melting method could allow fast evaluation of thermodynamic parameters.

When we applied established thermodynamic analysis to fluorescence melting data, we encountered problems that have not been solved in published literature. Melting profiles exhibited non-linear baselines, which were difficult to analyze. Oligonucleotide duplexes did not melt in two-state fashion and their transition enthalpies, entropies, and free energies were not in agreement with UV optical melting data. Some problems can be attributed to changes in fluorescence that takes place when temperature or pH are altered. We report here solutions to these issues that are encountered in fluorescence melting experiments of nucleic acids and offer a new avenue to extract thermodynamic energies from melting profiles.

## MATERIALS AND METHODS

Oligodeoxynucleotides were synthesized using phosphoramidite chemistry at Integrated DNA Technologies and purified by denaturing polyacrylamide gel electrophoresis or high-pressure liquid chromatography.^19^ All nucleic acid samples were at least 90% pure when purity was assessed by capillary electrophoresis (Beckman P/ACE MDQ system, Beckman Coulter, Fullerton, CA).^19^ DNA identity and purity was also confirmed by mass spectrometry using Oligo HTCS system (Novatia, Princeton, NJ). Experimentally measured and predicted molecular masses differed less than 2 g mol^−1^ for all oligodeoxyribonucleotides. Three studied dyes (TET, HEX, and Alexa Fluor 546) have shed carboxylic or chlorine groups during electrospray ionization in the Oligo HTCS system; this resulted in additional species that were occasionally observed in their mass spectra. Oligonucleotides were dialyzed against 10 m*M* Tris-HCl, 0.1 m*M* Na_2_EDTA buffer (pH 7.5) for at least 30 h (28-well microdialysis system, Gibco/BRL) at 5°C and stored in −20°C. Under these conditions, no degradation of modified oligonucleotides was detected in a year using capillary electrophoresis. Concentrations of DNA strands were determined from absorbance^20^ using extinction coefficients predicted from the nearest-neighbor model.^21^ Extinction coefficients of dyes and quenchers at 260 nm were taken into account (see Table S1 of Supporting Information).

When DNA concentrations are less than 300 n*M*, the composition of solutions can be adversely affected by adsorption of oligonucleotides to surfaces of plastic tubes. Hydrophobic chemical modifications, including dyes and quenchers, facilitate this interaction. We have therefore diluted samples to low DNA concentrations immediately prior conducting melting experiments. Adsorption tendencies of DNAs were evaluated for low-binding microcentrifuge tubes from several manufacturers. Both Costar tubes (Cat. No. 3207, Corning, Wilkes Barre, PA) and Marsh/Abgene non-stick tubes (Cat. No. 50T6050G, ABgene USA, Rochester, NY) were found to exhibit the lowest DNA adsorption. For long-term storage, concentrated DNA solutions (>50 μ*M*) were stored in screw capped O-ring tubes because snap-cap microcentrifuge tubes were not effective in preventing spontaneous water evaporation and loss of sample volume. No significant evaporation was seen in storage because our DNA solutions did not increase UV absorbance. Labeled oligonucleotides were stored in the dark and their exposure to light was limited as much as possible to avoid photobleaching.

### Melting Studies

Since most published thermodynamic parameters have been determined in 1*M* Na^+^ solution, we have also used a similar buffer containing 1*M* NaCl, 10 m*M* sodium phosphate, 1 m*M* Na_2_EDTA adjusted to pH 7.0 with NaOH.^20^ Complementary single-strands were combined in 1:1 molar ratio, heated to 95°C and slowly cooled to room temperature for ∼30 min to ensure formation of equilibrium structures. DNA samples were loaded into 96-well plate (25 μL per well), spun at 660 rcf for 2 min and equilibrated at starting temperature (5°C) for at least 5 min. Temperature dependence of fluorescence was measured at 200 n*M* concentration of dye-labeled single strands. Duplexes were melted at 13 single strand concentrations (*C*_t_) of 19, 30, 46, 70, 110, 160, 250, 375, 570, 870 n*M*, and 1.3, 2.0, 3.0 μ*M*. These values were designed to give uniformly separated data points on ln *C*_t_ scale. Each concentration was measured on an individual plate. It is not advised to measure different dye concentrations on the same plate because the iQ5 system automatically adjusts gain setting and collection time based on fluorescence of the brightest well. Fluorescence intensity was recorded every 0.2°C using iQ5 real-time PCR system (Bio-Rad laboratories, CA) that had a tungsten-halogen lamp source. The system had five optical filters; for each dye, we selected the filter recommended by iQ5 manufacturer. The iQ5 system was calibrated for well factors, background, and dye fluorescence signals at least every 3 weeks. Two heating and two cooling melting profiles were collected at the rate of 20–30°C h^−1^, which was sufficiently slow to establish equilibrium conditions. The protocol is shown in Figure S1 of the Supporting Information. Melting data for each DNA sample were processed with the Bio-Rad iQ5 Optical System Software (version 2.0). Values were averaged over at least two wells. We obtained reproducible *T*_m_ measurements (±0.4°C) using the Extreme Uniform 96-well thin wall plates (Cat# B70501, BIOplastics BV, Landgraaf, Netherlands). The *T*_m_ errors were up to two times larger when regular clear or black PCR plates were used.

Ultraviolet melting experiments were performed on a single beam Beckman DU 650 spectrophotometer, Micro *T*_m_ Analysis accessory, a Beckman High Performance Peltier Controller, and 1-cm pathlength quartz cuvettes (Beckman Coulter, Fullerton, CA) as previously described.^20^ Spectrophotometer was controlled by custom macro to more finely control the rate of temperature changes and to improve resolution. Absorbance values at 268 nm were measured every 0.1°C. UV experiments were conducted at *C*_t_ concentrations of 2 and 4 μ*M*. Both heating and cooling melting profiles were recorded for each DNA sample in two different cuvettes and temperature was increased linearly at a rate of 25°C h^−1^.

### Analysis of Melting Profiles

Fluorescence and UV melting profiles were analyzed using published procedures.^13^,^14^,^20^ Background fluorescence of plate wells was subtracted automatically by iQ5 software. We have programmed Visual Basic for Applications software in Microsoft Excel to analyze large sets of melting curves acquired by iQ5 real-time PCR system. Linear sloping baselines were automatically selected.^22^ The selections were reviewed and adjusted if the software would not choose proper baselines. The extent of melting reaction^13^–^15^,^20^ was described by fraction θ, which was calculated from fluorescence of DNA sample (*F*), fluorescence of upper baseline (*F*_U_), and fluorescence of lower baseline (*F*_L_) at each temperature, θ = (*F* − *F*_L_)/(*F*_U_ − *F*_L_). The value of θ depends on dissociation and distance between fluorophore and quencher. If duplex melting transition proceeds in a two-state (all-or-none) manner, i.e., partially melted duplexes are negligible throughout the melting transition, then θ will reflect the fraction of melted duplexes.^13^ This is also true for θ obtained from UV absorbance melting experiments. Melting profiles of θ versus temperature were smoothed^23^ and *T*_m_ values were determined as the temperature where θ = 0.5. Melting temperatures were averaged over all heating and cooling experiments. The average standard deviation of experimental melting temperatures was estimated to be 0.4°C.

Thermodynamic values of Δ*H*^o^, Δ*S*^o^, and Δ*G*^o^ were determined using two established methods that assume two-state melting transitions.^13^,^14^ First, the annealing constants for single strand-duplex equilibrium (*K*_a_) were calculated at each temperature, *K*_a_ = 2(1 − θ)/(θ^2^*C*_t_), for each melting profile. These equilibrium constants were least-squared fitted to van't Hoff relationship,


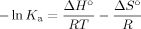
(1)

where *R* is the ideal gas constant. The Δ*H*^o^ and Δ*S*^o^ values were estimated from slopes and intercepts of fitted straight lines of ln *K*_a_ versus 1/*T* plots. Melting data where θ ranged from 0.15 to 0.85 were used in these fits. Thermodynamic values were averaged over studied DNA concentrations, heating and cooling melting profiles.

In the second method,^14^ Δ*H*^o^, Δ*S*^o^, and Δ*G*^o^ values were evaluated from the dependence of melting temperatures on DNA concentrations. The reciprocal values of average melting temperatures were plotted against ln *C*_t_ and fitted to linear relationships,


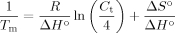
(2)

If thermodynamic values determined from both methods differ significantly, assumption of two-state nature for duplex melting transition is likely invalid.^14^

### Effects of pH and Quenchers on Fluorescence

Steady-state fluorescence of labeled single stranded oligodeoxynucleotides was measured in buffers of various pH containing 1 m*M* Na_2_EDTA and 20 m*M* citrate (pH = 5), or 20 m*M* Na_2_HPO_4_ (pH = 6, 6.5, 7, 7.5), or 20 m*M* Tris-HCl (pH = 8, 8.5, 9) at 25°C. PTI fluorescence spectrophotometer (Photon Technology International) with dual monochromators, R928 photomultiplier, and 75W Xenon Short Arc lamp was employed. Bandwidth was set to 4 nm. Analysis was done using Felix software (v1.4) supplied by manufacturer.

Abilities of Iowa Black, Black Hole, and Dabcyl quenchers to suppress dye fluorescence were studied in 1*M* Na^+^ melting buffer and at 25°C. Steady-state fluorescence signal (RFU_ss_) at the wavelength of emission maximum (Table I) was acquired for 200 n*M* solution of single stranded oligonucleotide (200 μL) where a fluorophore was attached to 5′ terminus. Five times molar excess of complementary oligodeoxynucleotide that contained a quencher at 3′ terminus was then added (∼1–3 μL). Remaining fluorescence was recorded every minute until a steady value (RFU_Q_) was obtained, which took from 2 to 5 min. Background fluorescence signal of the buffer was subtracted from fluorescence of DNA samples. Quenching efficiency (%) was calculated as 100 × [1 − (RFU_Q_/RFU_ss_)].

**Table I:** Average Properties of Various Dyes Attached to 5′ End of Two Single Stranded Sequences, CGTACACATGC and ACCGACGACGCTGATCCGAT

Fluorescent Dye	Excitation Maximum (nm)	Emission Maximum (nm)	Change of Fluorescence from 25 to 90°C
Weak temperature dependence			
Alexa Fluor 594	592	616	−12%
Texas Red	599	615	−14%
TET	524	535	−15%
ROX	587	605	−16%
MAX	533	562	−22%, −10%^a^
Alexa Fluor 546	557	571	−19%
HEX	536	553	−20%
Moderate temperature dependence			
FAM	494	520	+25%, −11%^a^
Bodipy 630/650	638	653	−37%
Alexa Fluor 488	494	517	−37%
Strong temperature dependence			
Rhodamine Green	504	531	−61%
Alexa Fluor 532	528	552	−64%
TAMRA	559	583	−67%
Cy5	648	667	−83%
Tye665	647	665	−85%
Cy3	549	565	−91%
Tye563	549	563	−92%

a Fluorescence change is significantly sequence dependent.

## RESULTS

**FIGURE 1:**
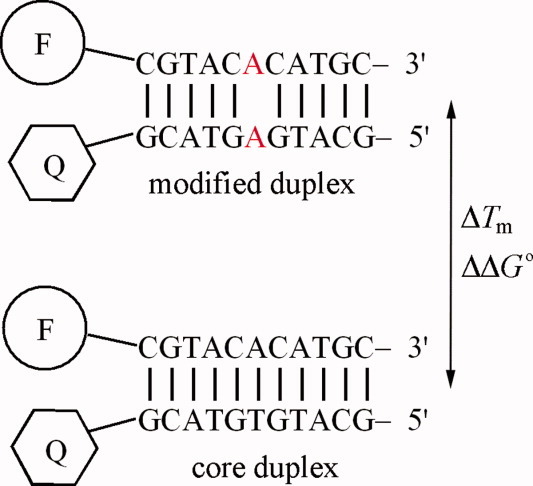
Thermodynamic impact of duplex perturbation (e.g., mismatch, bulge, chemical modification) is determined from the stability difference between modified and core DNA duplexes. Both duplexes contain the same fluorophore (*F*)–quencher (*Q*) pair, so that stabilizing effects of dye–quencher pair cancels out and does not significantly influence differential thermodynamic values (ΔΔ*G*^o^, Δ*T*_m_).

We have investigated suitability of fluorescence melting experiments to determine accurate thermodynamic values for DNA duplex denaturation. Fluorescence melting data could be used to quantify effects of structural perturbations or chemical modifications on DNA duplex stability from Δ*G*^o^ difference between the perfectly matched (core) and mismatched duplexes. An example is shown in Figure 1 where free energy change attributed to an A-A mismatch is determined. Similar schemes could be utilized for other DNA duplex perturbations (e.g., bulges, internal loops, dangling ends, chemical modifications). Using fluorescence signal instead of traditional ultraviolet absorbance would significantly speed-up data collection because fluorescence intensity for hundreds of duplexes could be monitored simultaneously using commercially-available real-time PCR equipment. Since vast majority of published fluorescence melting experiments employed 6-carboxyfluorescein (FAM) dye in combination with carboxytetramethylrhodamine (TAMRA) or Dabcyl quenchers, we first studied these dye-quencher combinations. Non-two-state nature of melting transitions was detected. When the same duplex samples were melted under the same solution conditions, we have observed significant differences between thermodynamic values (Δ*H*^o^, Δ*S*^o^, and Δ*G*^o^) obtained from UV and fluorescence experiments (see below). Because of this poor performance, we have examined a set of 22 commercially available dyes and quenchers. The ability of each dye-quencher pair to reliably report fine details of melting transitions was studied in order to find the optimal pair for fluorescence melting experiments. The ideal fluorophore should be inexpensive and yield high fluorescence values; exhibit negligible dependence of fluorescence on temperature and pH; be photostable when repeatedly heated and cooled, and exposed to light of the intensity encountered in real-time PCR equipment^24^; be efficiently quenched so that high signal to background ratio is achieved; show little interactions with, or quenching by, nucleobases; provide thermodynamic values that agree with UV melting experiments; and should not alter character of melting transition, so that the reaction becomes non-two-state.^13^,^14^

### Fluorescence of Dyes is Temperature and pH-Dependent

**FIGURE 2:**
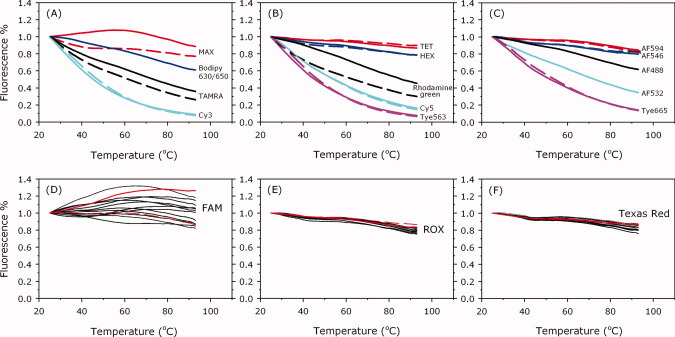
Dependence of fluorescence on temperature is shown for various dyes attached to 5′ end of single stranded oligonucleotides, CGTACACATGC (solid lines), ACCGACGACGCTGATCCGAT (dashed lines). Fluorescence is normalized to 100% at 25°C. “AF” is an abbreviation for Alexa Fluor dyes. Beside these two sequences (red lines), fluorescence was also measured for more than dozen other 5′ labeled sequences (black lines) in panels (D), (E), and (F).

Table I summarizes properties of commonly used fluorophores that were studied. Fluorescence of many dyes was found to vary with temperature. Temperature-dependent effects are more complex for dyes covalently attached to nucleic acids than for dyes alone because nucleobases can quench dyes. Both intrinsic fluorescence and quenching of dyes with neighbor nucleotides varies with temperature. Table I and Figure 2 show that Cy3, Cy5, Tye563, Tye665, TAMRA, Alexa Fluor 532, and rhodamine green attached to single-stranded oligonucleotides dramatically decrease fluorescence with increasing temperature. Such significant loss of signal makes analysis of fluorescence melting data difficult because the size of fluorescence change with temperature is comparable to changes of fluorescence seen upon duplex denaturation.

Results for FAM, MAX, Cy3, Cy5, Tye563, Tye665 dyes also reveals that their fluorescence varies with temperature in non-linear fashion. Analysis of melting profiles encompasses subtraction of linear baselines to calculate fraction of melted base pairs (see Materials and Methods). Non-linear dependence of dye fluorescence on temperature makes the linear baseline selection unreliable. Dependence of *F*_U_ and *F*_L_ on temperature would have to be experimentally determined. This may not always be possible and the multiple number of melting experiments would be necessary to analyze thermodynamic values for a single duplex sample.^25^,^26^

Figure 2 also identifies oligonucleotide-dye conjugates whose fluorescence does not change much with temperature. Texas Red, carboxy-X-rhodamine (ROX), hexachlorofluorescein (HEX), tetrachlorofluorescein (TET), and Alexa Fluor 594 are fluorophores that exhibited the favorable properties, i.e., their fluorescence only slightly decreased with increasing temperature and the change of fluorescence was approximately linear.

To further study effects of oligonucleotide sequence, we have measured temperature dependence of fluorescence for over dozen of different single-stranded sequences available in our lab, where FAM, ROX, or Texas Red were attached to 5′ terminus. Figures 2E and 2F reveal that Texas Red and ROX variation of fluorescence with temperature is consistent and independent of oligonucleotide base sequence. Figure 2D demonstrates that FAM temperature dependence of fluorescence varies widely and is unique for each oligonucleotide sequence.

**FIGURE 3:**
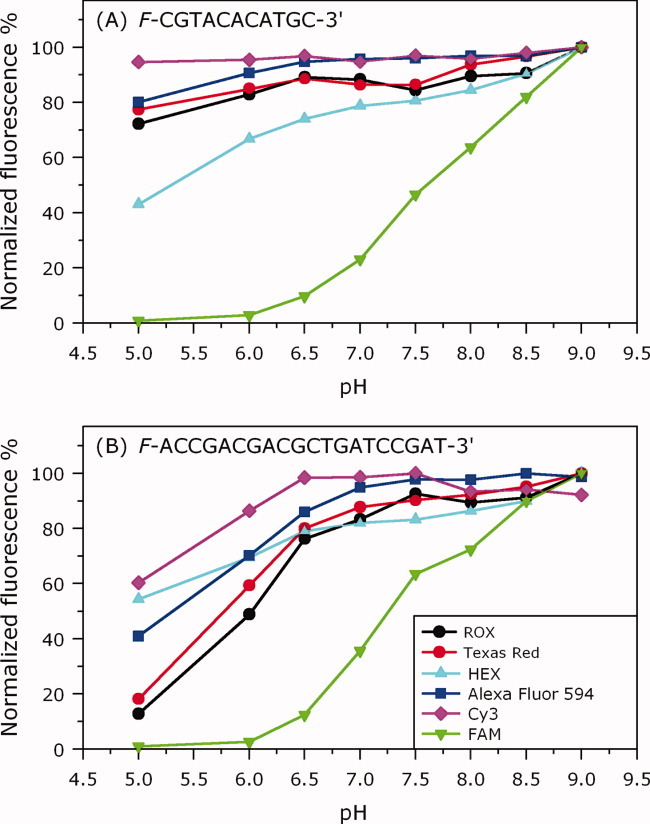
Dependence of fluorescence on pH at constant temperature (25°C). Various dyes were attached to 5′ terminus of two single stranded oligodeoxynucleotides: (A) CGTACACATGC, (B) ACCGACGACGCTGATCCGAT. Experimentally measured values were connected with straight lines to illustrate trends.

We have next studied acid/base equilibria of dye-oligonucleotide conjugates. Protonation or deprotonation of dyes alter their electronic structure, which in turn changes quantum yield and ability to fluoresce. Protonation of neighbor nucleobases alters their electron-donating properties that determine nucleotide quenching abilities.^27^ Our most relevant measurements are presented on Figure 3 where various dyes were attached to two different single stranded sequences. Each dye-oligonucleotide conjugate exhibits its unique dependence of fluorescence on pH. Trends of dependence on pH are both sequence-dependent and dye-dependent. Most of dye-labeled oligodeoxynucleotides showed generally stable fluorescence signal (changes less than 10%) in the pH range from 6.5 to 7.8. The FAM is a noticeable exception. In acidic pH solutions, the FAM and the other dyes based on fluorescein moiety (TET, HEX) decreased significantly fluorescence signal in agreement with previous reports.^28^,^29^ Oligonucleotides labeled with Cy3, rhodamine green, or Alexa Fluor dyes showed different behavior. Their fluorescence intensity was stable over a wide pH range (from 5.5 to 8.0). We next compared ability of various quenchers to diminish fluorescence of these dyes.

### Quenchers

Fluorophores can be quenched by direct contact with a quencher (static, contact quenching),^30^,^31^ or by dynamic quenching, i.e., fluorescence resonance energy transfer (FRET) over distance of several nanometers.^2^ We have measured quenching efficiencies of terminally labeled 11-mer and 20-mer duplexes where either static or FRET quenching dominated. Detail results are presented in Table S2 of the Supporting Information. Higher quenching efficiencies (>96%) were achieved using contact quenching than FRET quenching (<88%). Previous study of FAM-TAMRA pair on the opposite ends of eight base pair duplex reported comparable FRET quenching efficiency (63%).^2^

To obtain the largest change of fluorescence upon melting and low background noise, fluorophores and quenchers should therefore be incorporated at the same end of the duplex, as shown in Figure 1. We were able to measure reproducible fluorescent melting profiles for such duplexes down to ∼20 n*M* oligonucleotide concentrations. This arrangement has another advantage. One of the termini does not contain any attached labels, so perturbations could be introduced there. Thermodynamic effects could be determined for terminal mismatches, dangling ends, and coaxial stacking interactions.

Comparison of various quenchers also showed that Iowa Black RQ and Black Hole quenchers provided the highest quenching efficiency (98–99%). The Dabcyl and Iowa Black FQ quenchers were less effective (96–98%). Texas Red, ROX, and Alexa Fluor 594 dyes were quenched more efficiently than TET and HEX dyes.

### UV and Fluorescence Melting Experiments

**FIGURE 4:**
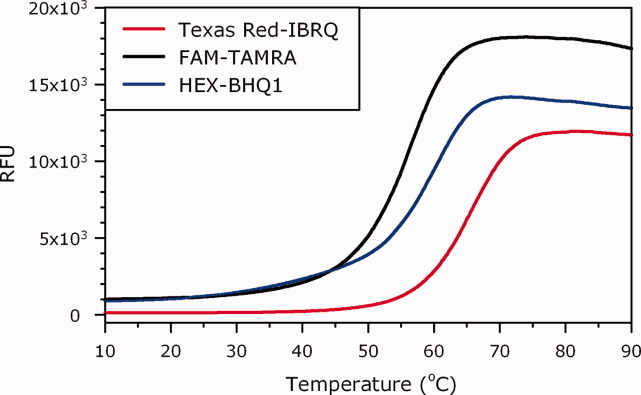
Average fluorescence melting profiles for three Seq1 duplexes where various dye–quencher pairs are attached at the terminus (*C*_t_ = 2 μ*M*).

Our next goal was to verify thermodynamic and thermal values determined from fluorescence melting experiments and compare them with UV melting data. Melting experiments were performed for DNA duplexes labeled with Texas Red, ROX, HEX, TET dyes and Iowa Black RQ or Black Hole quenchers. Alexa Fluor 594 was not studied because yields after synthesis and purification were lower than needed. We also investigated two commonly used combinations, FAM-TAMRA and FAM-Dabcyl pairs. Table II lists sequences of four studied duplexes. The dyes and the quenchers were attached to their termini. Three duplexes matched perfectly; the last duplex contained a single G-A mismatch. Using Bio-Rad iQ5 real-time PCR system, fluorescence melting profiles were acquired in the range of DNA concentrations from 19 n*M* to 3 μ*M*. Because of the detector limitations, fluorescent signal was noisy for HEX, TET, and FAM duplexes at *C*_t_ concentrations below 30 n*M* and we were unable to determine accurate *T*_m_ under those conditions. All duplexes exhibited single S-shaped melting profiles (see Figure 4). Since heating and cooling curves overlapped (data not shown), thermodynamic equilibrium conditions were achieved. The same duplexes were also melted using a UV spectrophotometer at 2 and 4 μ*M* DNA concentrations. Table III reports *T*_m_ values for various dye–quencher combinations. For each sample, the essentially same melting temperatures within the experimental error (±0.4°C) were obtained from fluorescence and UV melting experiments.

**Table II:** Sequences of Four Duplexes Employed in the Thermodynamic Study of Various Dyes

Name	Duplex Sequence^a^
Seq1	*F*-CGTACACATGC-3′/5′-GCATGTGTACG-*Q*
Seq2	*F*-CATACTACAAATA-3′/5′-TATTTGTAGTATG-*Q*
Seq3	*F*-ACTCGGTAGG-3′/5′-CCTACCGAGT-*Q*
Seq4^b^	*F*-ACTCGGTAGG-3′/5′-CCTAACGAGT-*Q*

a Fluorophore (*F*) and quencher (*Q*) were attached at duplex terminus.

b Bases of G-A mismatch are underlined.

**Table III:** Comparison of Melting Temperatures Between Fluorescence and UV Melting Experiments When Various Dyes and QuenchersWere Attached at Duplex Terminus

	Seq1			Seq2		
		
*F*–*Q*	Fluor. *T*_m_ (°C)^a^	UV *T*_m_ (°C)	Δ*T*_m_	Fluor. *T*_m_ (°C)^a^	UV *T*_m_ (°C)	Δ*T*_m_
Texas Red – IBRQ	64.9	65.3	−0.4	59.2	58.7	0.5
Texas Red – BHQ1	63.1	62.9	0.2	57.7	57.7	0.0
ROX – IBRQ	66.5	67.0	−0.5	61.7	61.5	0.2
TET – IBRQ	59.3	60.0	−0.7	54.6	54.2	0.4
HEX – IBRQ	59.5	60.5	−1.0	54.9	54.6	0.3
FAM – TAMRA	55.9	55.4	0.5	51.0	50.6	0.4
FAM – Dabcyl	55.2	54.8	0.4	50.2	50.0	0.2

a Melting temperatures determined from fluorescence experiments at *C*_t_ of 2 μ*M*. The IBRQ and BHQ1 are symbols for Iowa Black RQ and Black Hole Quencher 1, respectively.

**FIGURE 5:**
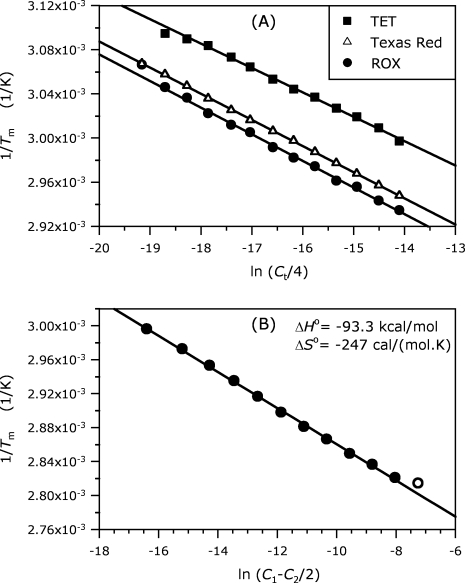
Plots of reciprocal melting temperatures versus DNA concentrations that were used to determine Δ*H*^o^, Δ*S*^o^, and Δ*G*^o^ values. Various dyes and Iowa Black RQ quencher were attached to Seq1 duplex terminus. (A) Plots where single strands are mixed in 1:1 ratio. (B) A plot where concentration of Texas Red-labeled DNA strand is kept constant (*C*_2_ = 150 n*M*) and concentration of the complementary Iowa Black RQ strand (*C*_1_) varies from 0.150 to 700 μ*M*. Open symbol indicates the data point that has not been used in the linear fit.

**FIGURE 6:**
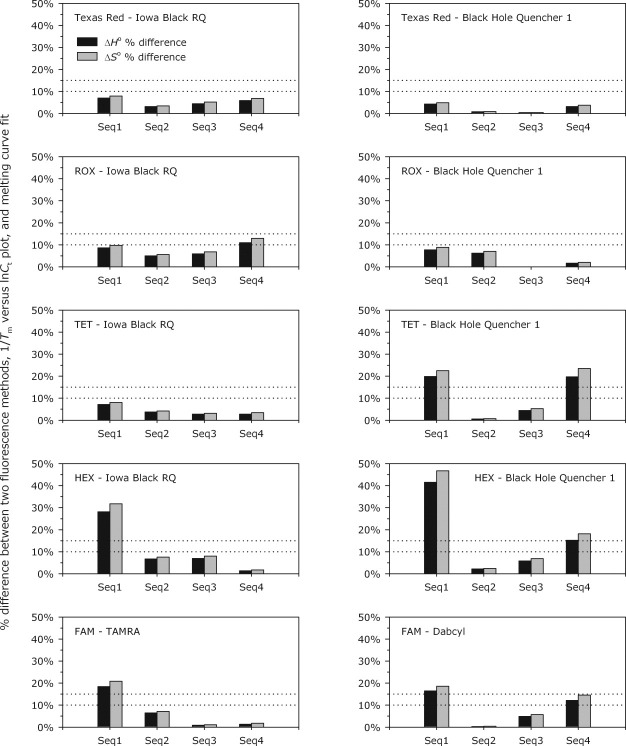
Comparison of thermodynamic values extracted from two fluorescence methods that are based on two-state assumption. Percent differences between transition enthalpies (black) or between entropies (gray) are plotted for four duplex sequences (see Table II). Differences larger than 15% imply non-two-state melting transitions. Label inside of each graph indicates a specific dye–quencher pair.

Next, we examined thermodynamic values. Transition enthalpies, entropies, and free energies were estimated from fits to melting profiles and from 1/*T*_m_ vs. ln *C*_t_ plots. Figure 5A shows examples of such plots generated from fluorescence melting data. Linear relationships were generally observed. The Δ*H*^o^, Δ*S*^o^, and Δ*G*^o^, values are presented in Table IV and Table S3 of Supporting Information. These thermodynamic values have been determined from fluorescence melting data assuming that short duplex DNAs melt in two-state manner and heat capacity change between these two states (duplex and random coil) is zero. If the two analytical methods described in Materials and Methods section, individual melting curve fit and 1/*T*_m_ vs. ln *C*_t_ plot, provide the same thermodynamic values within the experimental error (<15%), then two-state assumption is likely valid.^17^ Since these two methods depend differently on two-state approximation, significant disagreement indicates deviations from two-state model. Table IV and Figure 6 show differences in thermodynamic values between these two methods for various short DNA duplexes. Results reveal that differences between both methods are insignificant when duplex DNAs are labeled with Texas Red or ROX dyes. In contrast, substantial discrepancies are seen in enthalpies if FAM, HEX or TET dye is employed to monitor melting transitions. The Δ*H*^o^ values determined from 1/*T*_m_ vs. ln *C*_t_ plots are significantly more negative than the values obtained from melting curve fits and differ by more than 15% for these three dyes (see 4th column in Table IV). A similar level of discrepancy is observed for transition entropies. The findings suggest that these short FAM, HEX and TET duplexes do not melt in two-state fashion. Therefore, their thermodynamic values that were determined under two-state assumption are inaccurate.

**Table IV:** Comparison of Transition Enthalpies (kcal mol^−1^) for Seq1 DNA Duplexes Determined Using Fluorescence and UV Spectroscopy Methods

	Fluorescence			UV Spectroscopy	
		
Fluorophore (*F*) – Quencher (*Q*) Pair^a^	1/*T*_m_ vs. ln *C*_t_ Plot	Melting Curve Fit	% Difference^b^	Melting Curve Fit	% Difference^c^
Texas Red – IBRQ	−84.0	−90.0	6.9	−88.7	1.5
Texas Red – BHQ1	−84.0	−80.5	4.3	−81.9	1.7
Texas Red – BHQ2	−90.2	−79.7	12.4	−84.9	6.3
ROX – IBRQ	−82.4	−89.8	8.6	−87.0	3.2
ROX – BHQ1	−88.7	−82.1	7.7	−85.7	4.3
ROX – BHQ2	−90.7	−80.8	11.5	−86.1	6.4
TET – IBRQ	−89.8	−83.6	7.2	−91.6	9.1
TET – BHQ1	−97.3	−79.7	19.9	−89.7	11.8
TET – BHQ2	−99.1	−78.5	23.2	−90.2	13.9
HEX – IBRQ	−103.3	−77.8	28.2	−87.4	11.6
HEX – BHQ1	−111.5	−73.2	41.5	−91.6	22.3
HEX – BHQ2	−115.9	−73.6	44.6	−84.3	13.6
FAM – TAMRA	−96.4	−80.2	18.3	−89.2	10.6
FAM – Dabcyl	−98.0	−83.1	16.5	−88.0	5.7
FAM – BHQ1	−100.3	−80.6	21.8	−84.9	5.2

a Fluorophore and quencher were attached as shown in Figure 1. IBRQ, BHQ1, and BHQ2 are symbols for Iowa Black RQ, Black Hole Quencher 1, and Black Hole Quencher 2, respectively. Error of enthalpy values was estimated at 8%.

b Difference between two fluorescence methods.

c Difference between fluorescence and UV melting curve fit methods.

**FIGURE 7:**
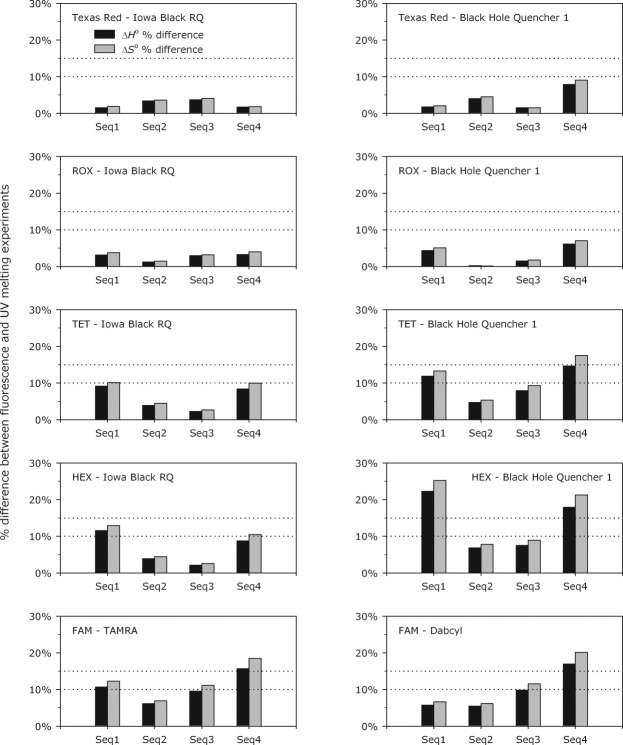
Comparison of ultraviolet and fluorescence melting experiments for four sequences (see Table II). Percent differences larger than 10% between Δ*H*^o^ values (black) or Δ*S*^o^ values (gray) suggest significant disagreement between ultraviolet and fluorescence methods. Label inside of each graph indicates a specific dye–quencher pair.

Table IV also presents comparison of transition enthalpies determined from the fluorescence and UV melting experiments. The Δ*H*^o^ values for Texas Red and ROX duplexes, which seemed to melt in two-state fashion, were in agreement between two spectroscopic methods (see the last column of Table IV). The significant differences (>10%) were seen for duplexes labeled with TET, HEX, and FAM dyes, which did not melt in two state manner. Similar results were obtained for transition entropies where TET, HEX, and FAM duplexes exhibited significant Δ*S*^o^ discrepancies between UV and fluorescence methods. Figure 7 summarizes those percent differences for four studied sequences and various dye–quencher combinations. In general, the differences in thermodynamic values between UV and fluorescence melting experiments are much larger for duplexes labeled with HEX, TET, and FAM dyes than for Texas Red and ROX oligonucleotides. When a duplex melts in two-state manner, agreement between fluorescence and UV melting experiments seems to be observed. These results also indicate that our melting curve fit and 1/*T*_m_ vs. ln *C*_t_ plots procedures are not appropriate for non-two-state melting transitions regardless of experimental melting method.

### Fluorophore and Quencher Affect Stability of Two Neighboring Base Pairs

The scheme displayed on Figure 1 assumes that there is negligible interaction between the fluorophore and the perturbation, e.g., A-A mismatch. If the terminal fluorophore or quencher alters thermodynamic effects of the perturbation, measured Δ*T*_m_ and ΔΔ*G*^o^ values would not reflect thermodynamic parameters for the perturbation in a native DNA sequence. To assess the number of base pairs whose stability is altered due to adjacent terminal labels, we carried out melting experiments for the set of mismatched duplexes (see Table V). The single base mismatch site was located from 1 to 10 base pairs away from the terminal fluorophore–quencher pair. First, the destabilizing effect of the mismatch was measured for the set of duplexes where Texas Red and Iowa Black RQ quencher were attached to the duplex terminus. Second, UV melting experiments were repeated for the set of the native duplexes of the same sequence where neither dye nor quencher were attached. The destabilizing effect of the same mismatch was compared between these two sets. We present melting temperature analysis in Table V because *T*_m_ values are robust and have low relative errors of measurements. The drop of *T*_m_ due to a terminal C-T mismatch was Δ*T*_m_(−) = −3.1°C when no labels were attached. Mismatch discrimination increased significantly, Δ*T*_m_(+) = −6.8°C, when Texas Red and Iowa Black RQ were attached next to the mismatch. As expected, dye and quencher moieties interacted with the nearest neighbor mismatch, so the mismatch discrimination observed using the labeled oligonucleotides does not agree with the mismatch discrimination observed in the native DNA duplex. When the mismatch site was introduced next to terminal base pair, again, significant difference between Δ*T*_m_(−) (−10°C) and Δ*T*_m_(+) (−14.5°C) is seen. However, when the mismatch is located three base pairs away from the labeled terminus, the dye and the quencher do not appear to influence the destabilizing effects of the mismatch. The Δ*T*_m_(+) and Δ*T*_m_(−) are essentially same (−10°C) for the T-T mismatch. These observations suggest that terminal fluorophore–quencher pair substantially affects stability of two adjacent base pairs. If a duplex perturbation is located farther away from the labeled terminus, the scheme on Figure 1 could be employed to determine thermodynamic parameters of the perturbation. A dye and a quencher attached at the terminus interact, form a complex and stabilize the duplex. However, in our design, stabilizing effects of dye–quencher complex are same for the core sequence and for the perturbed duplex, so dye–quencher pair does not affects the differential ΔΔ*G*^o^ and Δ*T*_m_ values determined using scheme on Figure 1.

**Table V:** Range of Terminal Dye–Quencher Interactions that Can Affect Nearby Mismatch Site Stability

			Texas Red-IBRQ Modification		No Modifications	
				
Sequence (5′ to 3′)^a^	δN_bp_^b^	Mismatch Pair	*T*_m_ (°C)^c^	Mismatch Δ*T*_m_(+)	*T*_m_ (°C)^c^	Mismatch Δ*T*_m_(−)
*F*-CGTACACATGC	—	None	65.3	—	49.1	—
*F*-CGTACACATGC	1	C-T	58.5	−6.8	46.0	−3.1
*F*-CGTACACATGC	2	G-A	50.8	−14.5	39.1	−10.0
*F*-CGTACACATGC	3	T-T	54.9	−10.4	39.0	−10.1
*F*-CGTACACATGC	4	A-A	55.7	−9.6	39.0	−10.1
*F*-CGTACACATGC	5	C-T	45.8	−19.5	31.3	−17.8
*F*-CGTACACATGC	6	A-A	54.0	−11.3	37.3	−11.8
*F*-CGTACACATGC	8	A-A	51.5	−13.8	34.4	−14.7
*F*-CGTACACATGC	10	G-A	59.2	−6.1	41.1	−8.0

a Mismatched base is underlined.

b Distance in base pair rise between terminal Texas Red - Iowa Black RQ modification and single base mismatch site.

c Melting temperatures were measured in 1*M* Na^+^ buffer and at total single strand concentration of 2 μ*M*.

### New Approach to Extract Thermodynamic Parameters from Fluorescence Melting Data

When duplexes shown in Figure 1 melt, fluorescence increases. This change of fluorescence is detectable even under conditions where the complementary quencher strand is present in vast excess. Therefore, any shifts of melting equilibrium and *T*_m_ values induced by additional amounts of quencher strand could be monitored by fluorescence. Equation (2) has been derived assuming that both single strands are present at the identical concentration (*C*_1_ = *C*_2_, *C*_1_+*C*_2_ = *C*_t_). When one strand is in excess (*C*_1_ > *C*_2_), the following relationships holds,^22^


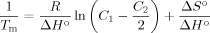
(3)

Melting temperatures could be measured for series of conditions where concentration of the fluorophore (*C*_2_) is kept constant and the quencher strand is added at increasing *C*_1_ concentrations. If 1/*T*_m_ is plotted against ln (*C*_1_ − *C*_2_/2), linear relationship is expected. Transition enthalpy and entropy can be determined from the slope and the intercept of this straight line. Equation (3) assumes that melting transitions are two-state and Δ*H*^o^, Δ*S*^o^ values are temperature-independent.

We acquired fluorescence melting data for DNA duplexes where the concentration of the strand labeled with Texas Red was kept constant (*C*_2_ = 150 n*M*) and concentrations of the complementary strand labeled with Iowa Black RQ varied from 150 n*M* to 700 μ*M*. The results are shown in Figure 5B. Fluorescent signal was found to be too noisy at the highest concentration probably because FRET quenching between unbound single strands became significant at high concentrations (the average distance between the dye and the quencher of melted strands was expected to be ∼50 Å). The remaining 11 data-points (*C*_t_ from 150 n*M* to 320 μ*M*) were least-square fitted to Eq. (3). Transition enthalpy of −93.3 kcal mol^−1^, transition entropy of −247 cal (mol K)^−1^ and transition free energy of −16.7 kcal mol^−1^ were obtained from the plot. These values are in agreement with experimental values determined using the established method of Eq. (2) (see the first row of Table IV, Δ*H*^o^ = −84 kcal mol^−1^, Δ*S*^o^ = −220 cal (mol K)^−1^ and Δ*G*^o^ = −15.9 kcal mol^−1^). The differences between methods are insignificant, less than 11%, which is within errors of Δ*H*^o^ and Δ*S*^o^ measurements for short duplex DNAs.^14^ We repeated this set of experiments for a duplex labeled with Texas Red–Black Hole Quencher 1 over the same range of concentrations (data not shown). Differences in thermodynamic values between new method [Eq. (3)] and established method [Eq. (2)] were again insignificant, less than 3%. Since new strategy allows measurements over wider range of DNA concentrations than 1/*T*_m_ vs. ln *C*_t_ method, it could be more accurate than the method based on Eq. (2).

## DISCUSSION

### Spectroscopic Properties of Dyes and Quenchers

We have studied suitability of fluorescence melting experiments to determine accurately fine details of DNA duplex thermodynamics using high-throughput real-time PCR systems. Procedures, practical considerations, strengths, weaknesses, and pitfalls generally encountered in fluorescence melting experiments have been discussed earlier^7^–^11^; the reader is referred to these excellent articles. Here, we are going to limit our discussion to new findings and applications of the method.

We have found 51 articles in the published literature that measured fluorescence-based melting profiles for duplexes, hairpins, triplexes, quadruplexes, and nanostructures labeled at termini with dyes and quenchers. Most of the articles restricted its analysis to melting temperatures and thermodynamic values were not determined. Over 85% of publications have utilized FAM fluorescence that was often quenched with TAMRA or Dabcyl moiety. While oligonucleotides modified with FAM-TAMRA pair have been preferred in the past because of easy synthesis, dozens of dyes and quenchers are now widely available and routinely conjugated to oligonucleotides.

Our results demonstrate that choice of dye and quencher is important. Since fluorescence of dyes is sensitive to microenvironment,^29^,^32^ effects of the oligomer being labeled on the spectroscopic properties of dye and quencher need to be considered. Fluorescence intensity changes when a dye is covalently attached to oligonucleotide because dyes are often quenched by neighbor bases. Buffer composition and pH affect fluorescence as well. These spectroscopic properties have been previously characterized for dyes based on fluorescein moiety.^28^ It has been demonstrated that FAM decreases its fluorescence in acidic pH and is quenched with neighbor guanine bases.^29^,^33^ The quenching mechanism usually involves electron transfer from the nucleobase ground state to the singlet excited state of the fluorophore.^27^ Torimura et al. collected Stern-Volmer plots of free FAM quenched with mononucleotides.^34^ Results revealed that both guanine and adenine diminish FAM fluorescence. TAMRA was strongly quenched by guanine as well. The same study also concluded that Texas Red does not appear to be significantly quenched by any base. We have seen that fluorescence of FAM-oligonucleotide conjugates is temperature-dependent and this dependence vary significantly with oligonucleotide sequence. Temperature affects quantum yield of dyes because nonradiative dissipation of energy from excited state is often enhanced with increasing temperature. Extinction coefficient of dyes changes with temperature as well. Both quantum yield and extinction coefficient will determine overall fluorescence signal.

Unruh et al.^29^,^35^ studied fluorescence, dynamics, and interactions of fluorescein, Texas Red, and TAMRA attached to an oligodeoxynucleotide. Texas Red fluorescence has been found to be insensitive to environment. Fluorescein moiety has shown fast rotational movements while Texas Red and TAMRA movements were slower and were dominated by the overall rotation of DNA molecule. These observations suggested that dianionic fluorescein is electrostatically repelled from negatively charged DNA surface, is relatively free to explore various conformations, and does not participate in stable stacking interactions. In contrast, zwitterionic Texas Red and TAMRA can bind to nucleotides. If they form stable interactions, their quenching with neighbor bases will be relatively steady until the oligonucleotide undergoes melting or annealing reaction.

DNA single strands do not behave exactly as a free random coil. Some level of base stacking and self-folding is expected, in particular, at low temperatures. When these semi-stable structures melt, average orientation and distance between FAM and neighbor guanines will be altered resulting in different amount of fluorescence quenching. The combined outcome of all these events is complex, non-linear dependence of FAM-oligonucleotide fluorescence with temperature that makes thermodynamic analysis of melting profiles difficult.

We have chosen Texas Red and ROX dyes for fluorescence melting experiments because they exhibit suitable spectroscopic properties. Their fluorescence is stable in pH range from 6.5 to 7.8, decreases slightly with temperature, and this change is linear. It is therefore possible to use linear baselines in analysis of melting profiles. Temperature dependence of their fluorescence is also independent of oligonucleotide sequence and presence of guanine. In agreement with our results, Nazarenko et al. observed that fluorescence of Texas Red is insensitive to GC base pair proximity.^33^ The Texas Red and ROX also exhibit good thermal and photo-stability; fluorescence intensity decreased less than 12% after two cooling and heating cycles (data not shown).

### Thermodynamic Values Determined from Fluorescence Melting Experiments

Thermodynamic parameters of nucleic acids have been traditionally determined using UV melting or differential scanning calorimetry experiments. New fluorescence melting experiments must provide the same results. To our knowledge, equivalence of thermodynamic values extracted from fluorescence and ultraviolet melting profiles for duplex DNAs has not been well established. Four published studies conducted limited comparison of thermodynamic values between both spectroscopic methods.^1^,^26^,^36^,^37^

Morrison and Stols have investigated 10 base pair long duplex 5′-TTG GTG ATC C-3′ modified with 5′ fluorescein.^1^ Its complementary sequence contained Texas Red moiety on 3′ terminus, which acted as a quencher. While melting temperatures were nearly identical between absorbance and fluorescence thermal experiments, enthalpies and entropies extracted from 1/*T*_m_ vs. ln *C*_t_ plots showed respective differences of 15 and 17%. Since the level of experimental uncertainties achievable at that time was high, fluorescence and absorbance melting profiles were concluded to be equivalent in spite of these discrepancies.^1^

Vámosi and Clegg studied UV and FRET-based melting profiles of 16 and 20 base pair long duplexes labeled with 5-carboxyfluorescein isothiocyanate and TAMRA on the opposite 5′ termini.^26^ They monitored the ultraviolet absorbance of DNAs, the fluorescence intensity of dyes, the fluorescence anisotropy of rhodamine, and the fluorescence energy transfer between dyes as a function of temperature. The helix-coil transitions were described well by the extended all-or-none model. Agreement between various methods was achieved when their analysis considered nonlinear character of baselines and substantial temperature dependence of TAMRA fluorescence. The differences of Δ*H*^o^ and Δ*S*^o^ values between fluorescence and UV melting data ranged from 7 to 13%.

Chen at al. have melted 5′-GTT TCA GTA TGA CAG CTG CGG-3′ duplex terminally labeled with Atto532 dye and Dabcyl quencher.^36^ Δ*H*^o^ values differed less than 4% between fluorescence and UV melting experiments for this sequence. Transition entropies were also in agreement. The differences increased to 15% when single G-A mismatch was introduced in the middle of the duplex indicating significant inconsistency between both methods. Thermodynamic values determined from individual melting profiles and 1/*T*_m_ vs. ln *C*_t_ plots were in agreement for the matched duplex, however, two-state assumption has not been investigated for the mismatched duplex.

Finally, Saccà et al. have measured melting processes of 4 × 4 tile nanostructures using FAM-TAMRA pair.^37^ UV experiments have sensed denaturation of the entire structure while the fluorescence method reported mostly thermodynamics of local double stranded arm to which fluorophores were attached. Therefore, it was not possible to directly compare experimental Δ*H*^o^ values between both spectroscopic methods; however, melting temperatures and extracted total Δ*H*^o^ value were roughly consistent between fluorescence and UV spectroscopy methods. Our systematic results are consistent with those findings and demonstrate uncertain performance of FAM-TAMRA pair in melting experiments. We observed Δ*H*^o^ and Δ*S*^o^ discrepancies up to 20% when FAM label was used. Texas Red or ROX probes showed better performance; the differences between UV and fluorescence methods were less than 9%.

When a fluorophore and a quencher are attached at the same duplex terminus, they are at close proximity and often interact to form a complex that leads to changes in absorbance spectrum,^19^ increases stability of DNA duplexes,^31^ and quenches fluorescence. Fluorescence melting profile reflects disruption of this complex; therefore, the signal will be most affected by opening of terminal base pair with attached labels. Ideally, the dissociation of dye–quencher complex is intimately connected with entire duplex denaturation, and both events occur simultaneously. This is likely to be the case for short duplexes (<16 base pairs) that melt in two-state (all-or-none) fashion.

Figure 6 shows that short duplexes labeled with HEX, TET, or FAM dyes exhibit significant discrepancies in their Δ*H*^o^, Δ*S*^o^ values suggesting deviations from two-state melting transitions. The HEX, TET, and FAM labels may induce deviations from two-state melting behavior or they may not faithfully report duplex DNA melting transitions. In such cases, thermodynamic values (Δ*H*^o^, Δ*S*^o^, and Δ*G*^o^) are questionable and may not be used to evaluate thermodynamic parameters of introduced duplex perturbation. It is necessary to establish validity of method assumptions to obtain reasonable thermodynamic values. We have observed that the same sequences labeled with Texas Red and ROX do not show such inconsistencies.

Figure 4 indicates that HEX and to lesser degree FAM oligonucleotides are showing non-linear “pre-melting” increase in fluorescence at temperatures below *T*_m_. This event makes melting curve fits to two-state model unreliable and leads to discrepancies between fluorescence and UV melting methods. Since such pre-melting transitions are not seen in UV melting profiles of the same HEX and FAM duplexes, we hypothesize that pre-melting transitions observed in these fluorescence melting curves reflect temperature dependent conformation changes and “loosening” of FAM-TAMRA and HEX-BHQ1 complexes while base pairs remain largely intact. Fluorescence method is expected to be more sensitive to deviations from two-state behavior than UV melting method. Origin of fluorescent signal is localized to duplex terminus while UV signal reflects absorbance of all nucleotides and is more likely to be proportional to the fraction of melted base pairs.

Others have also recognized inferior properties of fluorescein dyes for melting experiments. ATTO495 dye has been recently suggested as an viable alternative to FAM.^38^ Although thermodynamic information has not been determined, melting temperatures obtained using fluorescence were in agreement with *T*_m_ values determined by UV spectroscopy. The ATTO495 could be useful, in particular, in acidic buffers, but fluorophore also showed substantial decrease of intrinsic fluorescence with temperature. Texas Red and ROX do not exhibit such drawback.

### Effects of Terminal Labels on Stability of Neighboring Base Pairs

Data in Table V suggests that the stabilizing effect of terminal fluorophore–quencher pair is local and do not extend beyond two neighboring base pairs. This is consistent with short range of significant thermodynamic interactions observed in native duplexes. Nearest-neighbor model, which neglects interactions beyond neighboring base pairs, has been proven successful in predicting thermodynamics of DNA melting transitions. It should be noted that our experiments has been done in 1*M* Na^+^ environment. It is likely that at much lower salt (<70 m*M*), the range of significant interactions increases. For example, next-nearest-neighbor interactions in native DNAs have been found to be significant in 25 m*M* Na^+^.^39^

Several studies have examined range of fluorescein quenching by neighboring guanine bases. Nazarenko et al. observed quenching if at least one guanosine was present within four nucleotides from the FAM site.^33^ The similar effective range of interactions was reported for melting of 34 base pair long duplex labeled with fluorescein and TAMRA.^26^ Their statistical zipper model suggested that fluorescent signal is affected by the integrity of five base pairs in the vicinity of the dye. Unlike single base mismatches, other structural perturbations or modifications may have thermodynamic effects that extend beyond the nearest-neighbor base pair. To ensure that dye–quencher pair does not affect thermodynamics of duplex perturbation that is about to be measured, it is wise to introduce the perturbation site at least five base pairs away from the terminal dye–quencher pair.

### Hardware and Software

Real-time PCR systems were not designed for high-resolution thermodynamic experiments, so their ability to perform melting experiments varies widely. Most instruments have a choice of excitation and emission filters. The detector typically collects steady-state fluorescence integrated over the emission filter band. Measurements of anisotropy or fluorescence lifetime are not available. The ideal system would permit the temperature settings anywhere from 0 to 100°C in fine increments (0.1°C). The rate of temperature change needs to be slow enough to allow measurements under equilibrium conditions (most PCR equipment is intentionally designed to employ the fastest possible temperature ramp speeds). Fluorescence collection time must be added to calculate the overall rate of temperature change, which is sometimes neglected in the publish literature. For a given platform, if the available direct heating rates are too fast for equilibrium melts, one can set temperature in small steps as a “PCR cycle” and measure fluorescence once the temperature is equilibrated. The system should therefore allow several hundreds cycles. It is necessary to collect both heating and cooling melting profiles to ensure equilibrium conditions during melting experiments. Calibration and accuracy of temperature probes may vary between manufacturers of real-time PCR equipment.^40^ The temperature probe can be calibrated with small thermistors or by comparing *T*_m_ values of various standard samples between ultraviolet spectrophotometers and PCR systems. It has been reported that location of the well within the 96-well plate may have minor effects on experimental *T*_m_ values.^41^–^43^ We have achieved uniform and reproducible *T*_m_ results across wells. Slightly higher *T*_m_ error in outer wells than in inner wells was detected (Figure S2 of the Supporting Information). Inner wells are therefore preferred when very high accuracy of melting experiments is desired.

Real-time PCR systems also employ a lid heater that keeps the plastic cover of the sample plate at high temperature to prevent water condensation on the cover. When plate temperature is set below room temperature (<30°C), the lid heater may turn off, which can cause disturbances in fluorescence signal. This event can complicate analysis of melting profiles. If a PCR system allows user to control the lid heater, DNA samples that have low *T*_m_ (<35°C) are easier to measure.

Light source is also an important factor. Since Texas Red and ROX are excited by light in 580–600 nm range, argon lasers that supply light at 488 and 514 nm do not excite them well resulting in poor signal to background ratio. Tungsten-halogen or Xenon lamps are better because they supply broad, unstructured emission over wide range of wavelengths.

Baseline selection of melting profiles is impractical to do manually for hundreds of melting profiles a day. We recommend the second derivative algorithm^22^ that can select baselines automatically. The issues with automatic selection can be flagged for manual inspection by running replicates and by comparing *T*_m_ with *T*_max_ temperature where maximum of the first derivative of melting profile is located. The *T*_max_ values are expected to be 0.3–1.5°C larger^22^ than *T*_m_; differences outside of this range warrant careful inspection of melting profiles.

## CONCLUSION

We have demonstrated that accurate thermodynamic values can be obtained from fluorescence melting profiles of short duplex DNAs measured by real-time PCR systems. Since this method can provide thermodynamic values for hundreds of samples in a single melting run, it will allow fast determination of thermodynamic parameters. The Texas Red, ROX dyes and Iowa Black RQ, Black Hole quenchers are most suitable labels for fluorescence melting experiments. In future studies, we intend to employ the differential method shown in Figure 1 to quantify impacts of various chemical modifications and structural perturbations on duplex stability.
