# Supplementary material for: Measuring thermodynamic details of DNA hybridization using fluorescence
Source: Biopolymers. 2011 Jul;95(7):472–86. doi: 10.1002/bip.21615 (PMC3082624; doi:10.1002/bip.21615)
Supplement: Supplementary file 1 [file bip0095-0472-SD1.doc]

**Measuring Thermodynamic Details of DNA Hybridization Using Fluorescence**

Yong You, Andrey V. Tataurov, Richard Owczarzy

*Integrated DNA Technologies, 1710 Commercial Park, Coralville, IA - 52241*

(A)


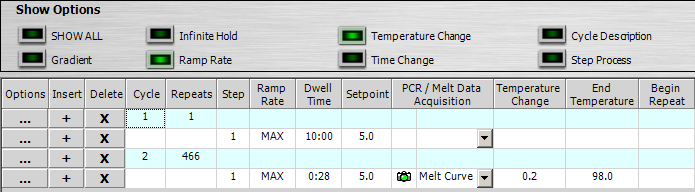


(B)


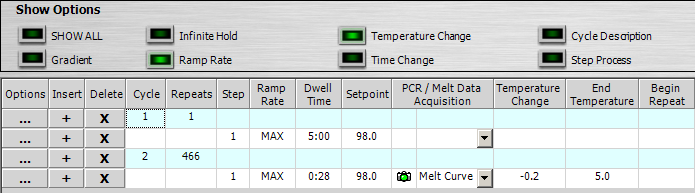


**Figure S1.** Step mode protocols for heating (A) and cooling (B) melting profiles in iQ5 real-time PCR software. Fluorescence was measured every 0.2^o^C from 5 to 98^o^C.

**Figure S2.** Well-to-well variations in melting temperatures were determined for 96-well plate. Fluorescent duplex, 5’‑ROX‑ACCGACGACGCTGATCCGAT/5’‑ATCGGATCAGCGTCGTCGGT-BHQ2, at *C*_t_ of 400nM and in 1M Na^+^ buffer was loaded to all wells. Experimentally measured melting temperatures from two heating and two cooling profiles were averaged over various sets of wells. (A) Melting temperatures in each well of 96-well plate. (B) Box represents the standard deviation of melting temperatures; mean *T*_m_ is indicated by the horizontal line in the middle of the box. The error bar shows the range from the minimum to maximum measured *T*_m_s. Thirty six outer wells are located in the first column 1, or the last column 12, or in the first row A, or in the last row H of the 96‑well plate. Sixty inner wells are inside of the plate, i.e., at intersections of columns 2-11 and rows B to G.

3’-Iowa Black RQ (Sp version) 5’-ROX

(/3IAbRQSp/) (/56-ROXN/)

5’-Texas Red (/5TexRD-XN/)

**Figure S3.** Chemical structures of quenchers at 3’ terminus and dyes at 5’ terminus.

**Table S1.** Extinction coefficients of dyes and quenchers that were used to predict extinction coefficients of modified oligonucleotides.

| Fluorescent dye or quencher | extinction coefficients at 260nm (L.mol^-1^.cm^-1^) |
| --- | --- |
| Alexa Fluor 488 | 41000 |
| Alexa Fluor 532 | 40600 |
| Alexa Fluor 546 | ~31000 |
| Alexa Fluor 594 | 43000 |
| Bodipy 630/650 | ~62000 |
| Black Hole Quencher 1 | 8000 |
| Black Hole Quencher 2 | 8000 |
| Cy3 | 4900 |
| Cy5 | 10000 |
| Dabcyl  (4-((4-(dimethylamino)phenyl)azo)benzoic acid) | 5580*^a^* |
| FAM (6-carboxyfluorescein) | 20960 |
| HEX (2’,4,4’,5’,7,7’-hexachlorofluorescein) | 31600 |
| Iowa Black FQ | 13340 |
| Iowa Black RQ | 44510 |
| MAX | 31200 |
| Rhodamine Green | 18000 |
| ROX (carboxy-X-rhodamine) | 22600 |
| TAMRA (carboxytetramethylrhodamine) | 29100 |
| TET (2’,4,7,7’-tetrachlorofluorescein) | 16300 |
| Texas Red | 14400 |
| Tye563 | 10000 |
| Tye665 | 35700 |

*^a^*Dabcyl is attached to a terminal dC nucleotide. This ε_260_ value does not include the extinction coefficient of dC, which is also added to the oligonucleotide sequence.

**Table S2.** Quenching efficiency (%) for various combinations of quenchers (*Q*) and dyes (*F*)

| Quencher (*Q*) | Fluorescent dye (*F*) | | | | |
| --- | --- | --- | --- | --- | --- |
|  | Texas Red | ROX | AF594*^a^* | HEX | TET |
|  | Contact quenching*^b^* | | | | |
| Iowa Black RQ | 99.5 | 99.4 | 99.7 | 98.0 | 98.5 |
| Black Hole Quencher 1 | 99.3 | 99.2 | 99.4 | 97.7 | 98.0 |
| Black Hole Quencher 2 | 99.2 | 99.1 | 99.4 | 98.0 | 98.1 |
| Dabcyl | 98.3 | 98.7 | 98.6 | 97.8 | 98.1 |
| Iowa Black FQ | 98.2 | 97.4 | 97.8 | 96.6 | 96.9 |
|  | FRET quenching, 11-mer*^c^* | | | | |
| Black Hole Quencher 2 | 75.8 | 80.4 | 68.1 | 87.8 | 84.9 |
|  | FRET quenching, 20-mer*^d^* | | | | |
| Black Hole Quencher 2 | 13.6 | 25.8 | 20.6 | 31.7 | 35.5 |

*^a^*Alexa Fluor 594.

*^b^*Values were averaged over two duplexes,

*F*-CGTACACATGC-3’ and *F*-ACCGACGACGCTGATCCGAT-3’

*Q*-GCATGTGTACG-5’ *Q*-TGGCTGCTGCGACTAGGCTA-5’

*^c^*Duplex *F*-CGTACACATGC-3’

3’-GCATGTGTACG-*Q*.

*^d^*Duplex *F*-ACCGACGACGCTGATCCGAT-3’

3’-TGGCTGCTGCGACTAGGCTA-*Q*’

**Table S3.** Average melting temperatures, transition enthalpies (kcal/mol), entropies (cal/(mol.K)) and free energies (kcal/mol) determined from fluorescence and UV melting experiments.

|  | Fluorescence | UV | Fluorescence | | UV | Fluorescence | | UV | Fluorescence | | UV |
| --- | --- | --- | --- | --- | --- | --- | --- | --- | --- | --- | --- |
|  |  |  | 1/*T*_m_ vs. | melting | melting | 1/*T*_m_ vs. | melting | melting | 1/*T*_m_ vs. | melting | melting |
|  |  |  | ln *C*_t_ plot | curve fit | curve fit | ln *C*_t_ plot | curve fit | curve fit | ln *C*_t_ plot | curve fit | curve fit |
| Duplex (*F*-*Q*)*^a^* | T_m_ (^o^C)*^b^* | T_m_ (^o^C)*^b^* | Δ*H*^o^ | Δ*H*^o^ | Δ*H*^o^ | Δ*S*^o^ | Δ*S*^o^ | Δ*S*^o^ | Δ*G*^o^_37_ | Δ*G*^o^_37_ | Δ*G*^o^_37_ |
| **Seq1 duplexes** | |  |  |  |  |  |  |  |  |  |  |
| Texas Red-IBRQ | 64.9 | 65.3 | -84.0 | -90.0 | -88.7 | -219.5 | -237.6 | -233.1 | -15.9 | -16.3 | -16.4 |
| Texas Red-BHQ1 | 63.1 | 62.9 | -84.0 | -80.5 | -81.9 | -221.2 | -210.6 | -215.0 | -15.5 | -15.2 | -15.3 |
| Texas Red-BHQ2 | 63.8 | 64.0 | -90.2 | -79.7 | -84.9 | -239.1 | -207.4 | -223.0 | -16.1 | -15.3 | -15.7 |
| ROX-IBRQ | 66.5 | 67.0 | -82.4 | -89.8 | -87.0 | -213.7 | -235.7 | -227.0 | -16.1 | -16.7 | -16.6 |
| ROX-BHQ1 | 65.1 | 64.7 | -88.7 | -82.1 | -85.7 | -233.5 | -213.7 | -224.9 | -16.3 | -15.8 | -16.0 |
| ROX-BHQ2 | 65.8 | 65.9 | -90.7 | -80.8 | -86.1 | -238.8 | -209.4 | -225.0 | -16.6 | -15.9 | -16.3 |
| TET-IBRQ | 59.3 | 60.0 | -89.8 | -83.6 | -91.6 | -241.4 | -222.6 | -246.2 | -14.9 | -14.6 | -15.3 |
| TET-BHQ1 | 57.7 | 58.3 | -97.3 | -79.7 | -89.7 | -265.5 | -211.7 | -241.8 | -14.9 | -14.0 | -14.7 |
| TET-BHQ2 | 58.8 | 59.3 | -99.1 | -78.5 | -90.2 | -269.9 | -207.3 | -242.4 | -15.3 | -14.2 | -15.0 |
| HEX-IBRQ | 59.5 | 60.5 | -103.3 | -77.8 | -87.4 | -282.1 | -204.8 | -233.0 | -15.8 | -14.3 | -15.1 |
| HEX-BHQ1 | 58.5 | 59.3 | -111.5 | -73.2 | -91.6 | -307.9 | -191.4 | -246.6 | -16.0 | -13.8 | -15.1 |
| HEX-BHQ2 | 59.7 | 60.5 | -115.9 | -73.6 | -84.3 | -319.9 | -191.8 | -223.9 | -16.7 | -14.1 | -14.9 |
| FAM-TAMRA | 55.9 | 55.4 | -96.4 | -80.2 | -89.2 | -264.5 | -214.6 | -242.7 | -14.4 | -13.6 | -13.9 |
| FAM-Dabcyl | 55.2 | 54.8 | -98.0 | -83.1 | -88.0 | -269.8 | -224.1 | -239.5 | -14.3 | -13.6 | -13.7 |
|  |  |  |  |  |  |  |  |  |  |  |  |
| **Seq2 duplexes** | |  |  |  |  |  |  |  |  |  |  |
| Texas Red-IBRQ | 59.2 | 58.7 | -90.4 | -93.2 | -90.1 | -243.1 | -251.7 | -242.8 | -15.0 | -15.2 | -14.8 |
| Texas Red-BHQ1 | 57.7 | 57.7 | -84.9 | -85.6 | -89.1 | -227.9 | -229.9 | -240.5 | -14.3 | -14.3 | -14.5 |
| ROX-IBRQ | 61.7 | 61.5 | -89.9 | -94.5 | -95.7 | -239.6 | -253.5 | -257.2 | -15.6 | -15.9 | -15.9 |
| ROX-BHQ1 | 61.1 | 60.8 | -96.6 | -90.8 | -90.6 | -260.3 | -242.7 | -242.4 | -15.9 | -15.5 | -15.4 |
| TET-IBRQ | 54.6 | 54.2 | -87.0 | -90.3 | -93.9 | -236.7 | -246.8 | -258.1 | -13.6 | -13.8 | -13.9 |
| TET-BHQ1 | 53.3 | 53.3 | -88.3 | -87.8 | -92.0 | -241.7 | -240.0 | -253.2 | -13.3 | -13.3 | -13.5 |
| HEX-IBRQ | 54.9 | 54.6 | -83.9 | -89.8 | -93.3 | -227.0 | -244.9 | -255.9 | -13.5 | -13.8 | -13.9 |
| HEX-BHQ1 | 54.6 | 54.1 | -85.5 | -87.3 | -93.5 | -232.0 | -237.6 | -256.9 | -13.5 | -13.6 | -13.8 |
| FAM-TAMRA | 51.0 | 50.6 | -89.4 | -95.4 | -101.4 | -247.0 | -265.4 | -284.4 | -12.8 | -13.1 | -13.2 |
| FAM-Dabcyl | 50.2 | 50.0 | -92.3 | -92.0 | -97.2 | -256.7 | -255.8 | -272.1 | -12.7 | -12.7 | -12.8 |

Table S3 (continued)

|  | Fluorescence | UV | Fluorescence | | UV | Fluorescence | | UV | Fluorescence | | UV |
| --- | --- | --- | --- | --- | --- | --- | --- | --- | --- | --- | --- |
|  |  |  | 1/*T*_m_ vs. | melting | melting | 1/*T*_m_ vs. | melting | melting | 1/*T*_m_ vs. | melting | melting |
|  |  |  | ln *C*_t_ plot | curve fit | curve fit | ln *C*_t_ plot | curve fit | curve fit | ln *C*_t_ plot | curve fit | curve fit |
| Duplex (*F*-*Q*)*^a^* | T_m_ (^o^C)*^b^* | T_m_ (^o^C)*^b^* | Δ*H*^o^ | Δ*H*^o^ | Δ*H*^o^ | Δ*S*^o^ | Δ*S*^o^ | Δ*S*^o^ | Δ*G*^o^_37_ | Δ*G*^o^_37_ | Δ*G*^o^_37_ |
| **Seq3 duplexes** | |  |  |  |  |  |  |  |  |  |  |
| Texas Red-IBRQ | 62.0 | 62.4 | -69.7 | -72.8 | -75.6 | -179.1 | -188.6 | -196.4 | -14.1 | -14.4 | -14.7 |
| Texas Red-BHQ1 | 60.5 | 61.2 | -68.4 | -68.7 | -69.7 | -176.2 | -176.9 | -179.7 | -13.8 | -13.8 | -14.0 |
| ROX-IBRQ | 64.8 | 65.3 | -74.8 | -79.3 | -81.7 | -192.3 | -205.9 | -212.6 | -15.1 | -15.5 | -15.8 |
| ROX-BHQ1 | 62.0 | 62.0 | -74.4 | -74.5 | -75.6 | -193.3 | -193.4 | -196.8 | -14.5 | -14.5 | -14.6 |
| TET-IBRQ | 59.3 | 59.0 | -73.5 | -75.5 | -77.2 | -192.2 | -198.3 | -203.7 | -13.9 | -14.0 | -14.0 |
| TET-BHQ1 | 55.6 | 55.3 | -71.9 | -68.8 | -74.4 | -190.0 | -180.2 | -197.8 | -13.0 | -12.9 | -13.1 |
| HEX-IBRQ | 60.2 | 59.8 | -70.8 | -75.9 | -77.5 | -183.5 | -198.9 | -204.0 | -13.9 | -14.2 | -14.3 |
| HEX-BHQ1 | 57.0 | 56.5 | -72.9 | -68.8 | -74.1 | -192.0 | -179.3 | -196.1 | -13.3 | -13.1 | -13.3 |
| FAM-TAMRA | 52.3 | 51.9 | -71.2 | -70.6 | -77.7 | -190.0 | -187.9 | -210.1 | -12.3 | -12.3 | -12.5 |
| FAM-Dabcyl | 51.8 | 51.0 | -74.0 | -70.5 | -77.8 | -199.2 | -188.0 | -211.1 | -12.3 | -12.2 | -12.3 |
|  |  |  |  |  |  |  |  |  |  |  |  |
| **Seq4 duplexes** | |  |  |  |  |  |  |  |  |  |  |
| Texas Red-IBRQ | 52.5 | 52.8 | -63.2 | -67.1 | -68.2 | -165.4 | -177.1 | -180.4 | -12.0 | -12.1 | -12.2 |
| Texas Red-BHQ1 | 50.3 | 50.7 | -64.1 | -62.1 | -67.2 | -169.5 | -163.3 | -178.7 | -11.5 | -11.5 | -11.8 |
| ROX-IBRQ | 56.1 | 56.5 | -65.5 | -73.1 | -70.8 | -170.0 | -193.5 | -185.9 | -12.8 | -13.1 | -13.1 |
| ROX-BHQ1 | 52.4 | 52.7 | -69.3 | -68.1 | -72.4 | -184.0 | -180.4 | -193.5 | -12.2 | -12.2 | -12.4 |
| TET-IBRQ | 50.5 | 50.1 | -66.3 | -64.4 | -70.1 | -176.0 | -170.1 | -187.9 | -11.7 | -11.7 | -11.8 |
| TET-BHQ1 | 46.1 | 45.9 | -70.3 | -57.7 | -66.8 | -191.9 | -151.6 | -180.6 | -10.8 | -10.7 | -10.8 |
| HEX-IBRQ | 51.4 | 50.8 | -64.5 | -63.6 | -69.4 | -170.0 | -167.1 | -185.6 | -11.8 | -11.8 | -11.9 |
| HEX-BHQ1 | 47.6 | 47.2 | -67.2 | -57.7 | -69.1 | -180.9 | -150.8 | -186.8 | -11.1 | -10.9 | -11.1 |
| FAM-TAMRA | 41.5 | 41.1 | -58.6 | -57.8 | -67.6 | -157.5 | -154.8 | -186.4 | -9.7 | -9.8 | -9.8 |
| FAM-Dabcyl | 41.3 | 40.9 | -64.8 | -57.4 | -68.1 | -177.6 | -153.6 | -187.9 | -9.7 | -9.8 | -9.8 |

*^a^*Table II shows base sequences. *^b^*DNA concentration, *C*_t_ = 2μM.
